# On the Radical Treatment of Certain Cases of Grave Concealed Accidental Hemorrhage

**Published:** 1893-02

**Authors:** 


ON THE RADICAL TREATMENT OF CERTAIN CASES OF GRAVE CONCEALED ACCIDENTAL HEMORRHAGE.—Storer, of Boston (*Boston Medical and Surgical Journal*, Vol. 77, No. 16), in considering the subject of concealed accidental hemorrhage as an emergency in obstetrical practice, has collected 153 cases. From these cases, of 126 children, ninety-four per cent. were stillborn or died shortly after birth; while of 152 mothers seventy-one died (46.7 per cent.). Of 142 cases of all varieties and degrees, in four craniotomy was done with four deaths; in sixty-three the threatment was expectant, with forty deaths; in nineteen version was done, with eight deaths; in five dilatation alone, with two deaths; in eighteen forceps were used, with four deaths; in seven ergot was given and nothing else done, with two deaths; in twelve the membranes were ruptured, with two deaths; in eight the membranes were ruptured and ergot given, with one death; in one Porro operation was done successfully; in five the membranes were ruptured and the os dilated without a death. In thirty-seven cases the hemorrhage occurred during labor; of these twenty-one died. In 105 cases it preceded the labor, with a mortality of forty-two. Fifty-seven per cent. of those treated expectantly died. The author recommends active interference in all cases, and favors the Porro operation, speedily executed, in those cases where the cervix is not dilated.—*University Medical Magazine.*

